# Phytohemagglutinin ameliorates HFD-induced obesity by increasing energy expenditure

**DOI:** 10.1530/JME-20-0349

**Published:** 2021-05-13

**Authors:** Yunxia Zhang, Jin Li, Hui-hui Wang, Jiao Li, Yue Yu, Bo Li, Li Huang, Changjing Wu, Xiaomeng Liu

**Affiliations:** 1 Institute of Neuroscience and Translational Medicine, College of Life Science and Agronomy, Zhoukou Normal University, Zhoukou, Henan, China; 2 College of Public Health, Xinxiang Medical University, Xinxiang, Henan, China

**Keywords:** CMAP, PHA, obesity, energy expenditure, BAT

## Abstract

Despite all modern advances in medicine, there are few reports of effective and safe drugs to treat obesity. Our objective was to screen anti-obesity natural compounds, and to verify whether they can reduce the body weight gain and investigate their molecular mechanisms. By using drug-screening methods, Phytohemagglutinin (PHA) was found to be the most anti-obesity candidate natural compound. Six-week-old C57BL/6J mice were fed with a high-fat diet (HFD) and intraperitoneally injected with 0.25 mg/kg PHA everyday for 8 weeks. The body weight, glucose homeostasis, oxygen consumption and physical activity were assessed. We also measured the heat intensity, body temperature and the gene expression of key regulators of energy expenditure. Prevention study results showed PHA treatment not only reduced the body weight gain but also maintained glucose homeostasis in HFD-fed mice. Further study indicated energy expenditure and uncoupling protein 1 (UCP-1) expression of brown adipose tissue (BAT) and white adipose tissue (WAT) in HFD-fed mice were significantly improved by PHA. In the therapeutic study, a similar effect was observed. PHA inhibited lipid droplet formation and upregulated mitochondrial-related gene expression during adipogenesis* in vitro*. UCP-1 KO mice displayed no differences in body weight, glucose homeostasis and core body temperature between PHA and control groups. Our results suggest that PHA prevent and treat obesity by increasing energy expenditure through upregulation of BAT thermogenesis.

## Introduction

The rising pandemic of overweight and obesity has received major attention worldwide. Obesity is a major cause for the development of debilitating conditions such as type 2 diabetes, cardiovascular disease, hypertension, and non-alcoholic steatohepatitis, cancer, all of which reduce life quality as well as lifespan (López-Suárez 2019). The obesity develops because of excessive food intake or inadequate total energy expenditure (TEE). Based on this, caloric restriction and increasing exercise are the most common way to prevent obesity for many people over a long period of time (Handschin 2016). Although these ways are effective, dieting and exercise must be maintained for a long time otherwise, the risk of obesity will regain. Meanwhile, bariatric surgery and anti-obesity drugs also have been used to treat obesity. Bariatric surgery is the most effective method to treat obesity and its complications. However, bariatric surgery still has its own risks and complexities (Thomas & Agrawal 2012, Bray *et al*. 2016). There are many anti-obesity drugs (pancreatic lipase inhibitors to reduce intestinal fat absorption, and anorectic to suppress appetite), including 2,4-dinitrophenol, orlistat, lorcaserin, phentermine/topiramate, naltrexone/bupropion, and liraglutide, are approved by the US Food and Drug Administration (FDA) (Daneschvar *et al*. 2016). However, several anti-obesity drugs have been withdrawn from the market due to the obvious side effects. For example, 2,4-dinitrophenol increases the risk of neurological diseases and cataracts (Daneschvar *et al*. 2016), and orlistat has some unacceptable side effects such as nephrotoxicity, hepatotoxicity, kidney stones, and pancreatitis (Weir* et al.* 2011). In recent years, natural compounds from plants have also been used for treating obesity. Cyanidin-3-glucoside (C3G) (You *et al*. 2017), arctigenin (Huang *et al*. 2012), rutin (Yuan *et al*. 2017), berberine (Christoffolete *et al.* 2004), capsaicin (Baskaran *et al*. 2016), resveratrol (Um *et al*. 2010), curcumin (Wang *et al*. 2015) and ginsenoside (Quan *et al*. 2020, Yao *et al*. 2020) could increase energy expenditure through the stimulation of thermogenic brown or beige adipocytes. However, more effective and safe candidates from plants are urgently needed to treat obesity.

Connectivity map (CMAP) includes a database and associated software that is produced by the Broad Institute and is composed of whole-genome gene expression profiles derived from human cell lines treated with various small molecules (Lamb *et al*. 2006, Qu & Rajpal 2012). The software can compare two sets of genes that are upregulated and downregulated in a specific condition with the whole CMAP database. CMAP enables a researcher studying a drug candidate, gene, or disease and compares its signature to the database to discover unexpected connections. Here, we used CMAP database to identify phytohemagglutinin (PHA) as one of the most promising candidates. PHA from *Phaseolus vulgaris* is a naturally existing glycoprotein (Bardocz *et al*. 1996). It is a mixture of different isolectins, including erythroagglutinin (PHA**-**E) and leukoagglutinin (PHA**-**L) (Wu & Sun 2012). PHA is a mitogen receptor of T-cell and stimulates T-cell proliferation to secret IL-1a and IL-6 (Ponomareva *et al*. 2012, He *et al*. 2019). PHA has been shown to inhibit human cancer cell proliferation and induce apoptosis (Kochubei *et al*. 2015). However, few studies regarding the effect of PHA on anti-obesity have been reported thus far. Thus, the study was designed to figure out whether PHA could ameliorate obesity and its related mechanisms in HFD**-**fed mice and C3H10T1/2 cells. We concluded that PHA could ameliorate obesity and had a previously unknown function of enhancing the whole-body metabolism by upregulating brown adipose tissue (BAT) function and beige formation in white adipose tissue (WAT), which could offer a therapeutic approach for obesity and its related diseases.

## Methods

### Connectivity map analysis

To obtain the obesity-related gene expression signature in WAT, we analyzed gene expression data from Gene Expression Omnibus (GEO) database (accession number: GSE123394) with GEO2R (https://www.ncbi.nlm.nih.gov/geo2r). Those genes were separated into up- and downregulated expression group. We used HomoloGene (NCBI Resource Coordinators, 2014) to convert the mouse gene identifiers for probe annotations to human gene identifiers, then selected the probes that matched the mouse**-**to**-**human converted identifiers on the HG-U133A chip. CMAP scores the similarity of the up- and down-lists with the expression patterns of microarray data in CMAP. As a result, enrichment scores are returned by the software (Liu *et al*. 2015*a*). The enrichment score obtained from CMAP for compounds is the measure of similarity between the up-and down-list provided to software, and the up- and downregulated genes in the whole microarray obtained from treatment with the compounds. It was then used to query the CMAP (Subramanian *et al*. 2017) to obtain score of compounds in the database.

### Chemicals

PHA was purchased from Sigma-Aldrich. Insulin, triiodothyronine powder, indomethacin, 3-isobutyl-1-methylxanthine, and dexamethasone were purchased from Sigma-Aldrich. MEM and fetal bovine serum were obtained from Gibco (Thermo Fisher Scientific).

### Animal model

Six**-**week**-**old C57BL/6J male mice were purchased from the Model Animal Research Center of Nanjing University (China). In a facility certified by the Laboratory Animal Welfare Department, three mice in each cage were housed under a 12 h light:12 h darkness cycle. Because PHA is a glycoprotein, in order to avoid being digested and decomposed in the intestinal tract, we treated mice by intraperitoneal injection of PHA dissolved in saline. Food and water were provided* ad libitum*. Mice were fed with a HFD (60 kcal% fat; D12492) and subjected to intraperitoneal injection administration 0.1, 0.2, 0.25, 0.5, 1.0 mg/kg/day body weight doses of PHA, respectively. Body weight results showed that the 0.25 mg/kg/day injection dose is the minimum working concentration.

Prevention study mice were fed with a HFD and subjected to intraperitoneal injection administration of 0.25 mg/kg body weight doses of PHA at 18:00 h everyday for 8 weeks. Control groups received saline of equal volume. There were 25 replicates for each group. The body weight was measured weekly. At the end of the experimental period, Blood from mice eyes was collected into tubes containing EDTA and protease inhibitors for triglyceride (TG), blood glucose and total cholesterol (TC) measurements. BAT and WAT isolated for gene expression and western blot analyses were rapidly collected, frozen in liquid nitrogen, and stored at −80°C. BAT and WAT isolated for hematoxylin and eosin (H&E) and immunohistochemistry were immediately treated with 4% paraformaldehyde.

Homozygous male UCP-1 KO mice (genetic background C57BL/6J)were purchased from Jackson Labs. Six-week-old male UCP-1 KO mice fed with a HFD were randomly assigned into two groups and were administered an intraperitoneal injection of 0.25 mg/kg/day of PHA for 8 weeks. The average weight was determined weekly. Glucose homeostasis was determined for mice after treatment with PHA or saline. All animals received care according to the China Council on Animal Care and all procedures were approved by the Health Sciences Animal Welfare Committee of Zhoukou Normal University.

### Assessment of glucose homeostasis

After intraperitoneal injection for 8 weeks, the glucose tolerance testing (GTT) was performed on 16 h-fasted mice (Aryal *et al*. 2018). Blood glucose was measured with an Accu-Chek glucometer (Roche Diagnostics Corp) at 0, 15, 30, 45, 60, 90 and 120 min after an intraperitoneally administered injection of glucose at 1.5 g/kg. The insulin tolerance testing (ITT) was performed on 4 h fasted mice (Hu *et al*. 2018). The glucose concentrations were measured by venous bleeding at 0, 15, 30, 45 and 60 min after an intraperitoneal injection of human insulin at 1.0 U/kg. TG and Cholesterol plasma levels were quantified by a homogeneous enzymatic colorimetric assay (Spinreact, S.A., Spain).

### Temperature measurements and infrared imaging of heat intensity measurements

Each mouse’s rectal temperature was measured by a rectal probe connected to a digital thermometer (Yellow Spring Instruments) after exposure to the cold chamber (4°C) for 4 h with free access to food and water during treatment with PHA treatment for 8 weeks. Infrared imaging of heat intensity in mice was recorded with an infrared camera (E60: Compact Infrared Thermal Imaging Camera; FLIR; West Malling, Kent, UK).

### Oxygen consumption and physical activity

Oxygen consumption and physical activity were determined for mice at 8-week treatment with either PHA or saline. Oxygen consumption measurements were performed using TSE lab master systems (TSE Systems, BadHomburg, Germany) (Chi & Wang 2011). All mice were acclimatized for 24 h prior to measurements, then the volume O_2_ was measured over the course of the next 24 h. Mice were maintained at 25°C under a 12 h light:12 h darkness cycle with free access to food and water. The physical activity of mice was measured by optical beam technique (Opto-M3; Columbus Instruments, Columbus, OH, USA) over 24 h and calculated as 24 h average activity.

### RNA isolation and quantitative real-time PCR

Total RNA from C3H10T1/2 cells, BAT and epididymal white adipose tissue (eWAT) was extracted using Trizol reagent (Invitrogen). The concentration and quality of RNA were assessed with a NanoDrop 2000 (Thermo) and agarose gel electrophoresis. One microgram of total RNA was used for RT with the PrimeScript RTreagent kit with gDNA Eraser (Takara). The quantitative real-time PCR (qPCR) reaction was performed in a LightCycler 96 (Roche) system using the Go Taq® qPCR Master Mix (Promega). The sequence of primers can be found in Supplementary Table 1 (see section on supplementary materials given at the end of this article). The Ct (2^−ΔΔCt^) method was used to analyze the relative gene expression data according to the literature.

### Western blot

Cells and tissues were lysed in RIPA buffer containing protease and phosphatase inhibitors according to the manufacturer's instruction (Beyotime, Jiangsu, China). Protein lysates were heated at 95°C for 5 min in 5× sodium dodecyl sulfate (SDS) sample buffer and were separated with SDS-PAGE (30 μg each lane). After electrophoresis, proteins were transferred to PVDF membranes (Millipore) using a Mini Trans-Blot Cell system (Bio-Rad). The membrane was blocked with 5% non-fatmilk for 1.5 h at room temperature. Then the membrane was incubated with primary antibody specific for UCP-1 (ab10983; Abcam) overnight at 4°C. The membrane was incubated with IgG-HRP-conjugated secondary antibodies for 1 h at room temperature. The membranes were visualized by ECL (Bio-Rad).

### H&E and immunohistochemistry

Fixed tissues were sectioned after being embedded in paraffin. Sections with 5 μm thickness were stained with H&E then images were acquired by microscope. The mean area of adipocytes from each animal was calculated as previously described (Chen & Farese 2002). For immunohistochemical staining, BAT specimens were deparaffinized, boiled in sodium citrate buffer (10 mM sodium citrate, 0.05% Tween 20, pH 6.0) for 20 min, blocked with 5% normal goat serum for 60 min, incubated with anti-UCP-1 antibody (1:400 dilution; Cat. # ab10983; Abcam) at 4°C overnight and then incubated with the HRP-conjugated secondary antibody for 1 h at room temperature. UCP-1 signal was detected with DAB kit (ZSGB-BIO, Beijing, China) according to the manufacturer’s instruction and images were captured with an Olympus BX51 system.

### White and brown adipocyte differentiation

Mesenchymal precursor cells C3H10T1/2 (ATCC) were cultured in growth medium (MEM containing 10% fetal bovine serum, FBS). White adipocyte differentiation was induced by treating cells under basal adipogenesis conditions (MEM containing 10% FBS, 5 μg/mL insulin, 1 μM dexamethasone and 0.5 mM isobutylmethylxanthine, 100 μM indomethacin) for 2 days. The medium was then replaced by that supplemented with only insulin for another 4 days. The white adipocytes were treated with PHA (10 μM) or PBS during induction and differentiation period for 6 days. Then differentiated adipocytes used for Oil red O staining, RNA and protein extraction.

Brown adipocyte differentiation was induced by treating cells for 2 days under basal adipogenesis conditions (MEM containing 10% fetal bovine serum, 5 μg/mL insulin, 1 μM dexamethasone, 0.5 mM isobutylmethylxanthine, 120 μM indomethacin, and 1 nM 3,3,5-triiodo-L-thyronine (T3)). Then cells were switched to MEM containing 10% FBS only containing insulin and T3 for another 4 days. The brown adipocytes were treated with PHA (10 μM) or PBS during induction and differentiation period for 6 days. Then differentiated adipocytes used for Oil red O staining, RNA and protein extraction.

### Oil red O staining

Cells were fixed in 4% formaldehyde and stained with filtered Oil Red O for 10 min. Then the cells were washed with distilled water. Images were captured with an Olympus BX51 system.

### Animal model for therapeutic study

Therapeutic study mice were fed with a HFD for 8 weeks to induce obesity. Then, the HFD-induced mice subjected to intraperitoneal injection administration of 0.25 mg/kg body weight doses of PHA at 18:00 h every day for another 8 weeks. Control groups received saline of equal volume. There were 25 replicates for each group. The body weight was measured weekly. GTT, ITT and oxygen consumption were determined for mice of treatment with PHA. At the end of experimental period mice were fasted 16 h and killed by cervical dislocation. Blood was collected into tubes containing EDTA and protease inhibitors for determining the content of triglyceride TG and total cholesterol.

### Statistics

Data were analyzed using GraphPad Prism 7.0 software (Graphpad Prism, San Diego, CA, USA). Significant differences were determined using an unpaired, two-tailed student’s test (for comparison of two experimental conditions) or one-way ANOVA (for comparison of three or more experimental conditions). All values are presented as means ± s.e.m. (*P < 0.05, **P < 0.01, **P < 0.01). The number of animals used for each experiment is showed in the figure legends.

## Results

### Identification of PHA as a potential anti-obesity natural compound

**Figure 1:**
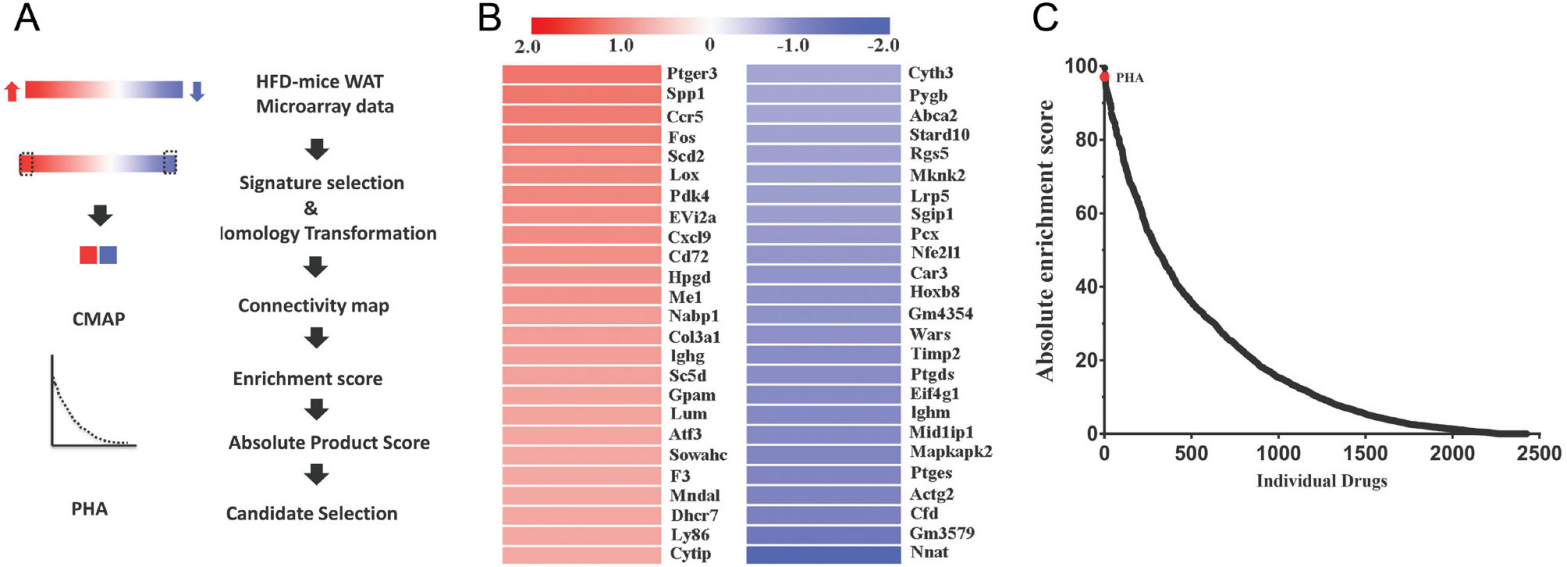
Identification of PHA as a potential ant-obesity natural compound. (A) Summary flow chart showing the identification of PHA as a potential anti-obesity candidate. (B) Heatmaps representing the selected 25 upregulated (red) and 25 downregulated (blue) genes in eWAT from obese mice. (C) Distribution of the calculated absolute enrichment score of individual small molecules, the red dot represents PHA. A full colour version of this figure is available at https://doi.org/10.1530/JME-20-0349.

Genetics play a major role in determining the obesity of HFD-fed mice (Coleman & Hummel 1973). Obesity is also accompanied by changes in gene expression. We hypothesized that compounds reversed the gene expression profile of HFD-fed mice would have an anti-obesity effect. To test this hypothesis, we referenced gene expression signatures by utilizing the microarray data obtained from WAT in mice with obesity (GSE123394) (Fig. 1A) (Almind & Kahn 2004). We chose the 25 genes that were most highly upregulated and another 25 genes that were most heavily downregulated in HFD mice (Fig. 1B), and we converted the mouse gene identifiers for our probe annotations to human gene identifiers. CMAP scores the similarity of the up- and down-lists with the expression patterns of microarray data in CMAP. At last, a total of 39 compounds with an absolute enrichment of more than 80 were identified, among which PHA is one of the most promising candidate compounds (Fig. 1C). Thus, we propose the PHA is a novel treatment option for obesity.

### PHA prevents HFD-induced obesity

**Figure 2:**
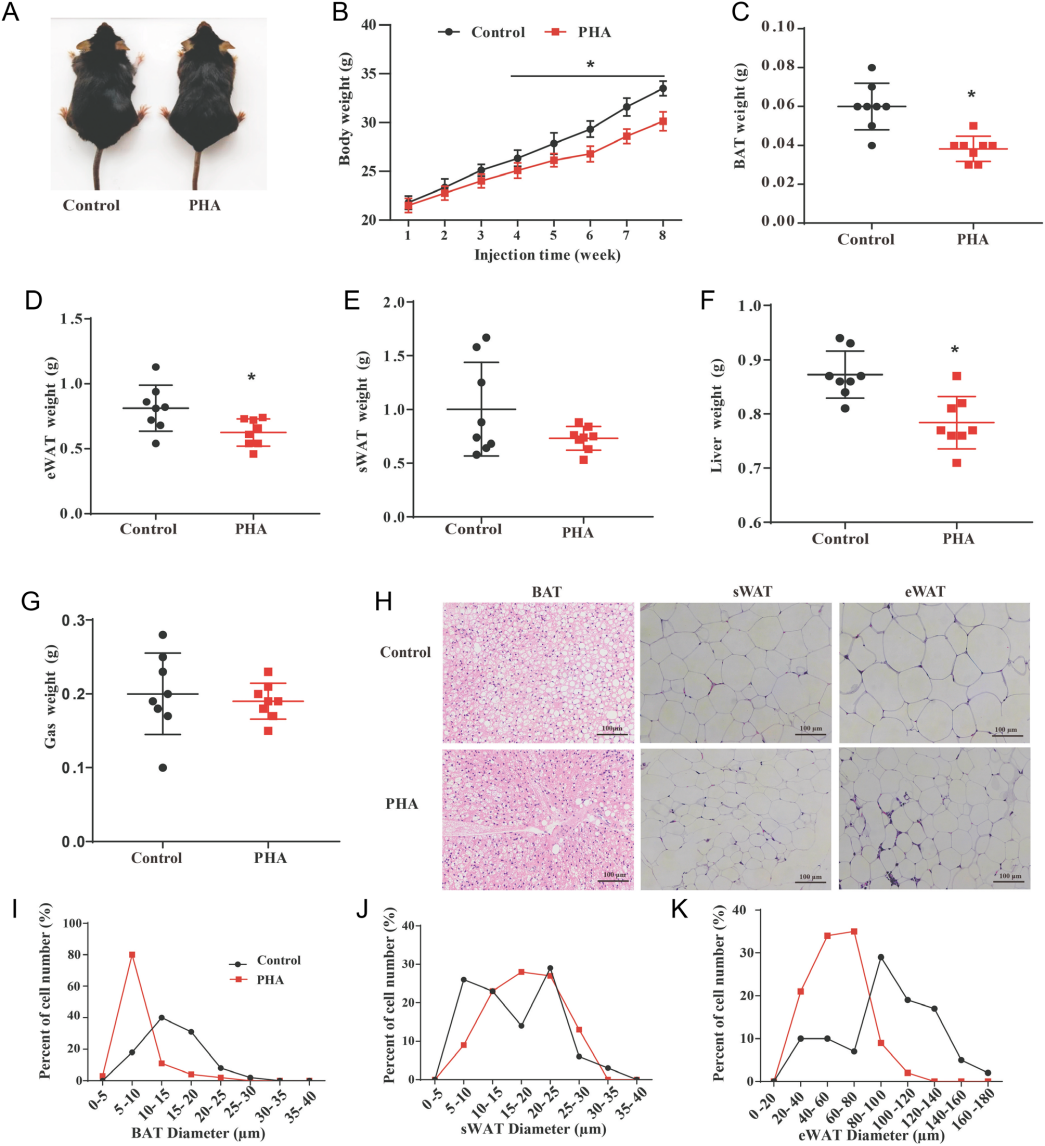
PHA reduces body weight gain of HFD-fed mice. (A) The size of HFD-fed mice with daily intraperitoneal injection of saline or PHA at a dose of 0.25 mg/kg for 8 weeks. (B) The body weight of HFD-fed mice with daily intraperitoneal injection of saline or PHA (*n* = 10 for each group). BAT (C), eWAT (D), sWAT (E), Liver (F), Gas (gastrocnemius) (G) weight of control and PHA group mice (*n* = 8 for each group). (H) H&E staining from BAT, sWAT and eWAT section, Scale bar, 100 µm. (I–K) The diameters of lipid droplets BAT (I), sWAT (J), and eWAT (K) Sections from control and PHA-treated mice. The data are presented means ± s.e.m. *P < 0.05. A full colour version of this figure is available at https://doi.org/10.1530/JME-20-0349.

To assess the ability of PHA to prevent the development of obesity, mice were fed with the HFD and were treated with PHA by intraperitoneal injection administration for 8 weeks. After treatment, no morphological and functional abnormalities were found in HFD mice. We found that PHA decreased body weight gain of PHA-treated HFD mice (Fig. 2A and B). In particular, from the fourth week until the end of treatment, PHA significantly reduced the body weight gain of HFD-fed mice (Fig. 2B). Then we isolated and weighed organs of PHA and saline-treated HFD-fed mice. The BAT, eWAT and liver weight of PHA-treated mice was significantly lower than that of the saline-treated group (Fig. 2C, D, E and F). However, PHA did not affect the mass of organs such as subcutaneous white adipose tissue (sWAT) (Fig. 2E), gastrocnemius (Gas) (Fig. 2G), kidneys, heart, and spleen after treatment (data not shown). The H&E staining showed that the size of the lipid droplets in eWAT and BAT of the PHA-treated mice was smaller than that of the control mice, whereas no significant effects on the size of the lipid droplets in sWAT (Fig. 2H, I, J and K). PHA treatment ameliorates HFD-induced obesity and affects adipose tissue composition in mice.

### PHA treatment improves glucose homeostasis and energy expenditure in HFD-fed mice

**Figure 3:**
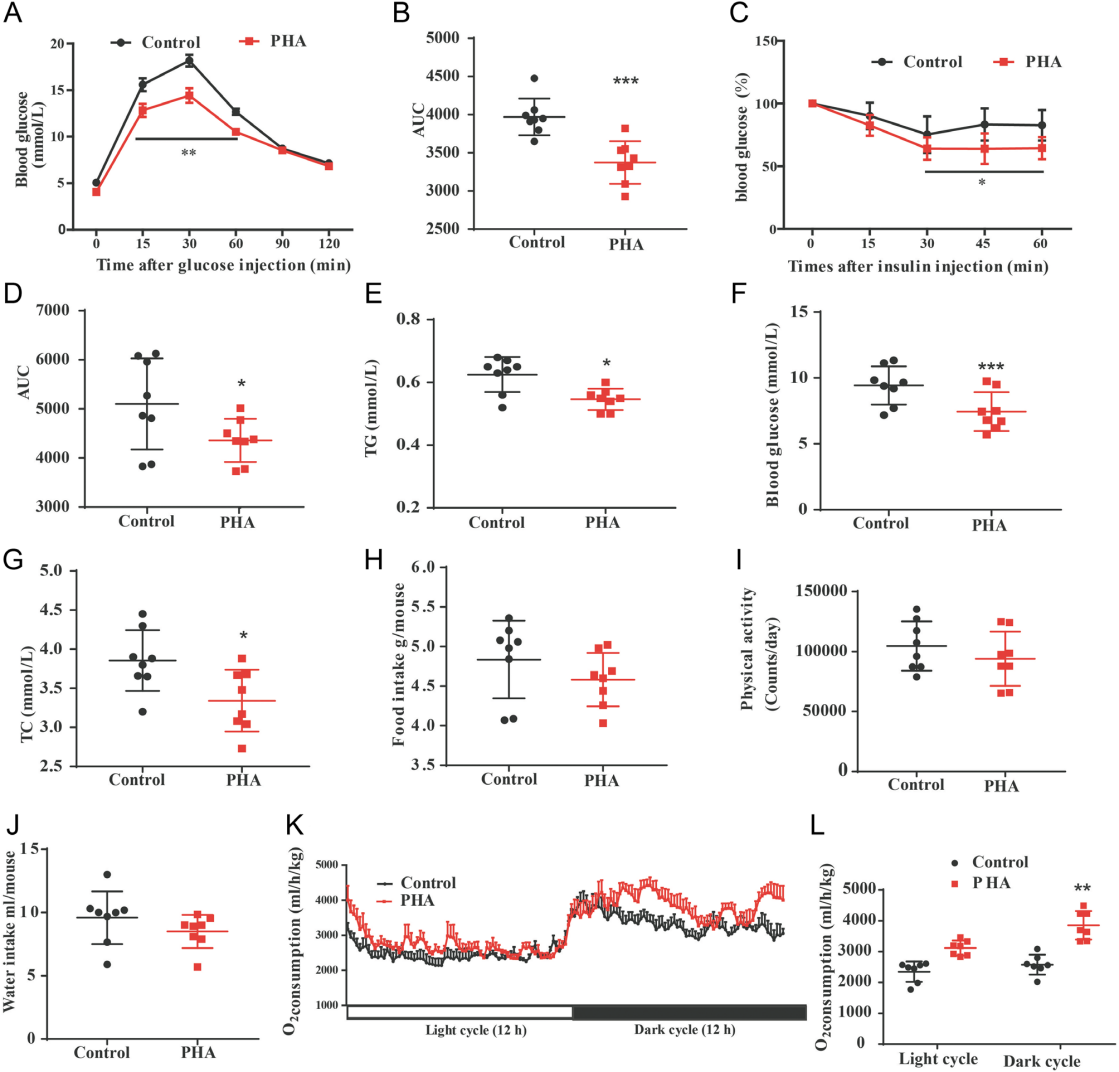
Effects of PHA treatment on glucose homeostasis and energy expenditure in HFD-fed mice. (A) GTT on control and PHA-treated HFD mice (injected with 1.5 g glucose per kg after overnight fast (*n* = 8 for each group) (B) Average area under the curve of GTT result. (C) ITT on control and PHA-treated HFD mice (*n* = 8 for each group). (D) Average area under the curve of ITT result. (E) TG levels in the plasma of control and PHA treatment mice (*n* = 8 for each group). (F) Blood glucose levels of control and PHA treatment mice. (G) TC levels in the plasma of control and PHA treatment mice (*n* = 8 for each group). (H) Daily food intake of control and PHA-treated HFD-fed mice during the fourth week of treatment (*n* = 8 for each group). (I) Physical activity during the fourth week of treatment (*n* = 8 for group). (J) Daily water intake during the fourth week of treatment (*n* = 8 for each group). (K) PHA treatment increased oxygen consumption during 24 h period in HFD-fed mice PHA treatment (*n* = 8 for group). (L) Scatter plot represent the average for each group. The data are presented means ± s.e.m. *P < 0.05, **P < 0.01, ***P < 0.001. A full colour version of this figure is available at https://doi.org/10.1530/JME-20-0349.

Clearance of glucose from the circulation during GTT was significantly faster in PHA-treated mice than in the control mice (Fig. 3A and B). ITT results suggested PHA also improved insulin sensitivity in HFD-fed mice (Fig. 3C and D). Serum profiles including TG, blood glucose levels and TC were also significantly reduced after PHA treatment (Fig. 3E, F and G). Adiposity often causes alteration of energy balance (Tseng *et al*. 2010). There were no significant differences in food intake, physical activity and water intake between PHA and control group mice (Fig. 3H, I and J). The PHA-treated mice showed markedly higher oxygen consumption during the 12 h darkness cycle than the control mice (Fig. 3K and L). This suggests that the PHA-treated mice consume more energy during active periods compared to the control mice.

### PHA enhances thermogenic program under HFD-fed by increasing BAT activity and promoting browning of WAT

**Figure 4:**
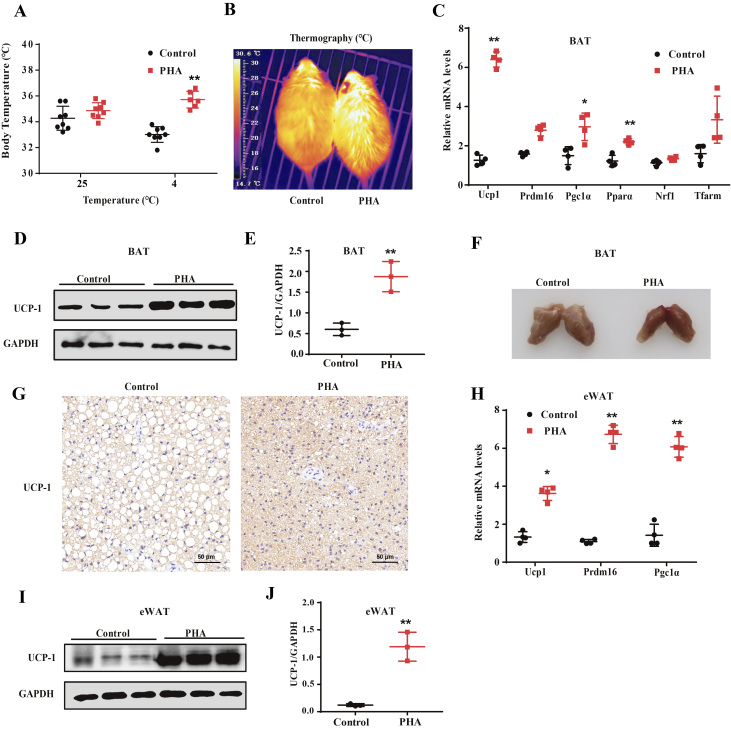
PHA enhanced heat production of BAT and browning of WAT. (A) Core body temperature of control and PHA-treated mice after 8 weeks of injection at room temperature (25°C) and 4°C for 4 h (*n* = 8 for each group). (B) Infrared thermal images shows BAT interscapular temperature after PHA treatment. (C) Gene expression profile in BAT, qPCR analysis of thermogenic-related gene, fatty acid oxidation-related gene and mitochondrial-related gene expression in BAT of control and PHA-treated mice after 8 weeks of injection (*n* = 4 for each group). (D) Western blot results of UCP-1 protein levels after PHA treatment in BAT of mice. (E) Relative protein expression levels represented by ratio of detected protein to GAPDH protein expression level in BAT of mice (*n* = 3 for each group). (F) Representative photography of BAT from saline-treated and PHA-treated HFD-fed mice after 8 weeks of injection. (G) Immunohistochemistry for UCP-1 protein (brown stain) in BAT sections of control and PHA-treated mice. Bars, 50 µm. (H) qPCR analysis of *Ucp1, Prdm16, Pgc1α* gene expression in eWAT of control and PHA-treated mice (*n* = 4 for each group). (I) Western blot results of UCP-1 protein levels after PHA treatment in eWAT of mice. (J) Relative protein expression levels represented by ratio of detected protein to GAPDH protein expression level in eWAT of mice. The data are presented as the mean ± s.e.m. (*n* = 3 for each group). *P < 0.05, **P < 0.01. A full colour version of this figure is available at https://doi.org/10.1530/JME-20-0349.

The heat production is one of the most important indicators of non**-**shivering thermogenesis in BAT. To further investigate the differences in energy expenditure between PHA-treated and control group mice, we conducted a cold tolerance test to evaluate the capacity of adaptive thermogenesis among the HFD-fed mice. Although there was no difference between PHA-treated and control groups at 25°C, PHA treatment greatly increased core body temperature when mice were exposed to a cold environment (Fig. 4A). The infrared imaging of heat intensity measurements also showed that PHA-treated HFD-fed mice could maintain higher temperature compared with control mice (Fig. 4B), which demonstrated PHA treatment significantly increased the thermogenic activity of brown fat in HFD-fed mice. Thus, we detected some genes expression though qPCR and found that the expression of *Ucp1*, proliferator-activated receptor α (*Pparα*) and PPARγ coactivator 1-alpha (*Pgc1a*) dramatically increased in BAT of PHA-treated HFD mice (Fig. 4C), whereas no significant differences were found on PR domain-containing protein *16* (*Prdm16*), mitochondrial transcription factors A (*Tfarm*) and nuclear respiratory factor-1 (*Nrf1*) expression. The results of Western blot and immunohistochemistry showed PHA treatment increased the expression of UCP-1 at the protein level in the BAT (Fig. 4D, E, F and G). The volume of BAT was decreased significantly and the color of BAT was not whitening in PHA-treated mice (Fig. 4F). 'Beige' cells in WAT, similar to brown adipocytes, also contains a high number of mitochondria and express BAT-specific genes (Rachid *et al*. 2015). Our qPCR results showed *Ucp1*, *Pgc1α* and *Prdm16* mRNA were significantly increased in eWAT of the PHA-treated group (Fig. 4H). UCP-1 protein level was also enhanced by PHA in eWAT (Fig. 4I and J). These results indicate that PHA can increase BAT activity and induce WAT browning.

### PHA inhibits white adipogenic differentiation and promotes brown adipogenic differentiation

**Figure 5:**
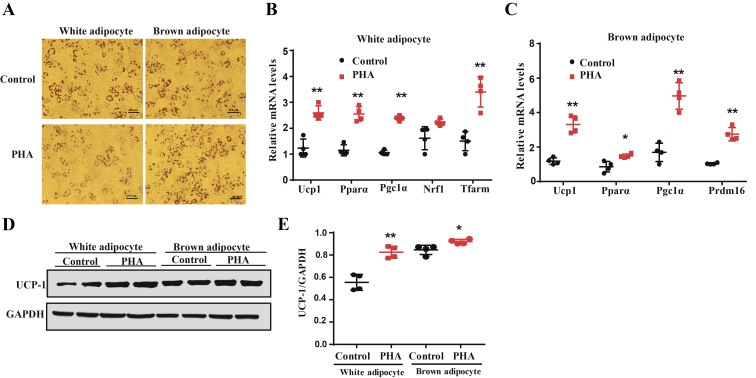
PHA inhibits white adipogenic differentiation and promotes brown adipogenic differentiationin C3H10T1/2 cells. (A) Lipid droplets were stained by oil red O. Bars, 100 µm. (B) qPCR analysis of thermogenic-related gene, fatty acid oxidation-related genes and mitochondrial-related gene expression profile in white adipocyte of control and PHA-treated group (*n* = 4 for each group). (C) qPCR analysis of thermogenic-related gene, fatty acid oxidation-related genes in brown adipocyte of control and PHA-treated group (*n* = 4 for each group). (D) Western blot results of UCP-1 protein levels after PHA treatment (*n* = 2 for each group). (E) Relative protein expression levels represented by ratio of detected protein to GAPDH protein expression level in white adipocyte and brown adipocyte. The data are presented as the mean ± s.e.m. *P < 0.05, **P < 0.01. A full colour version of this figure is available at https://doi.org/10.1530/JME-20-0349

C3H10T1/2 cells were induced to differentiate into white adipocytes or brown adipocytes while treated with PHA. PHA treatment appeared the low intensity of fat droplets during white adipogenic differentiation. In contrast, PHA treatment showed a high intensity of fat droplets during brown adipogenic differentiation (Fig. 5A). The expression of *Ucp1*, *Pparα*, and *Pgc1a* in PHA-treated mice was higher than those expressions in the control group during white and brown adipogenesis (Fig. 5B and C), and the expression of *Tfarm* was also increased by PHA during white adipogenesis (Fig. 5B). PHA treatment increased UCP-1 protein expression during both white and brown adipogenesis (Fig. 5D and E). Those results show that PHA inhibits white adipogenesis but promotes brown adipogenesis and white adipocyte browning.

### PHA does not prevent HFD-induced obesity in UCP-1 KO mice

**Figure 6:**
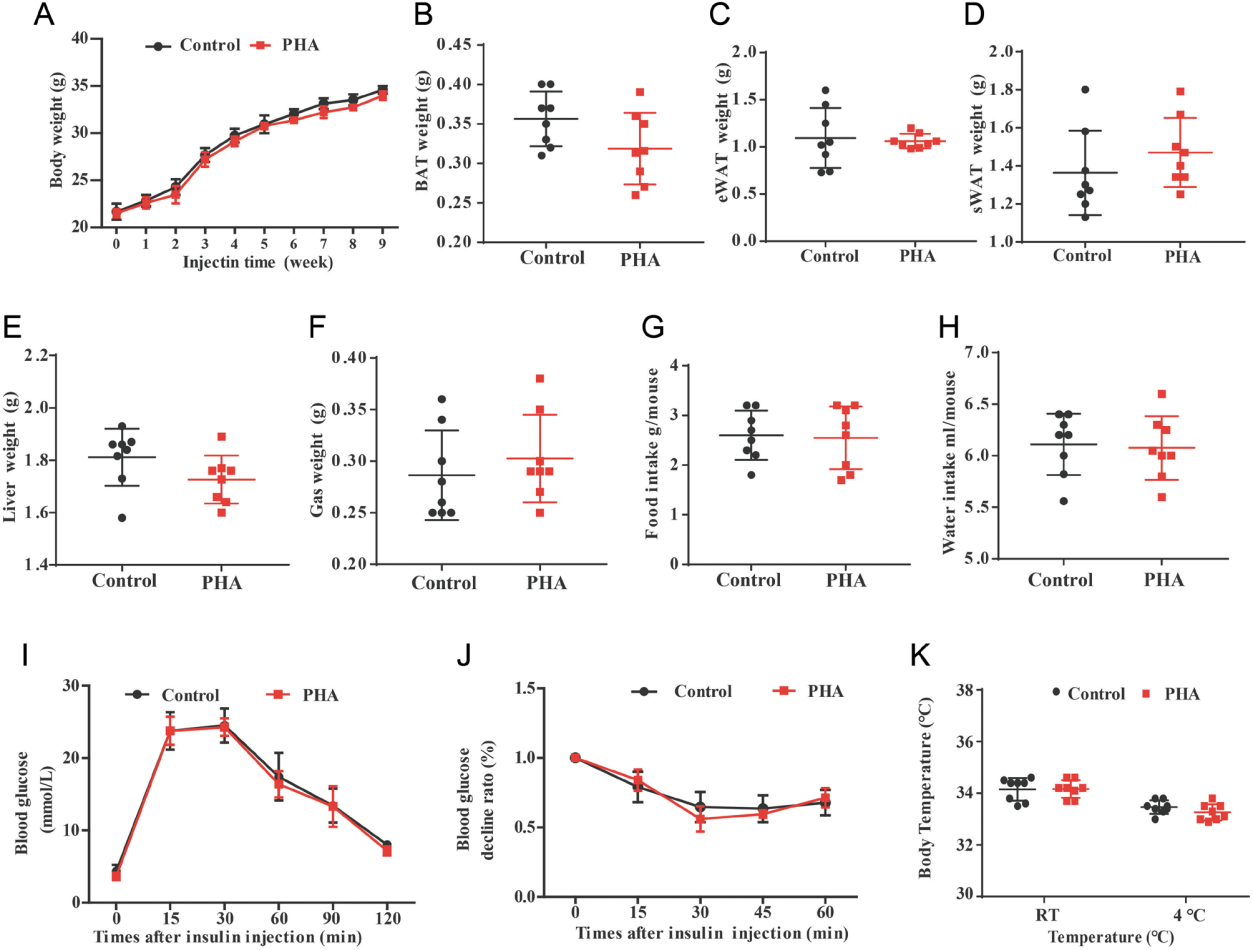
UCP-1 KO mice displayed no differences between PHA and saline groups when fed a HFD for 8 weeks. (A) The body weight of UCP-1 KO mice (*n* = 6 for each group). BAT (B), eWAT (C), sWAT (D), Liver (E), Gas (F) weight of UCP-1 KO mice (*n* = 8 for each group). (G) Daily food intake of UCP-1 KO mice (*n* = 8 for each group). (H) Daily water intake during the fourth week of UCP-1 KO mice (*n* = 8 for each group). (I) GTT on UCP-1 KO mice (J) ITT on UCP-1 KO mice (*n* = 8 for each group). (K) Core body temperature of UCP-1 KO mice after injection at room temperature (25°C) and 4°C for 4 h (*n* = 8 for each group). A full colour version of this figure is available at https://doi.org/10.1530/JME-20-0349.

To evaluate whether the PHA would prevent obesity by increasing BAT activity, we assessed the effects of PHA on obesity in UCP-1 KO mice. UCP-1 KO mice displayed no differences in body weight, body fat, liver and Gas weight between PHA and saline groups when fed a HFD for 8 weeks (Fig. 6A, B, C, D, E and F). There was no significant difference in food/water intake between PHA and control group mice (Fig. 6G and H). These results showed glucose tolerance, insulin sensitivity and core body temperature at both RT and cold environment had no difference in two groups of UCP-1 KO mice (Fig. 6I, J and K). These results indicate that PHA does not prevent HFD-induced obesity in UCP-1 KO mice.

### PHA has a therapeutic effect on HFD-induced obesity

**Figure 7:**
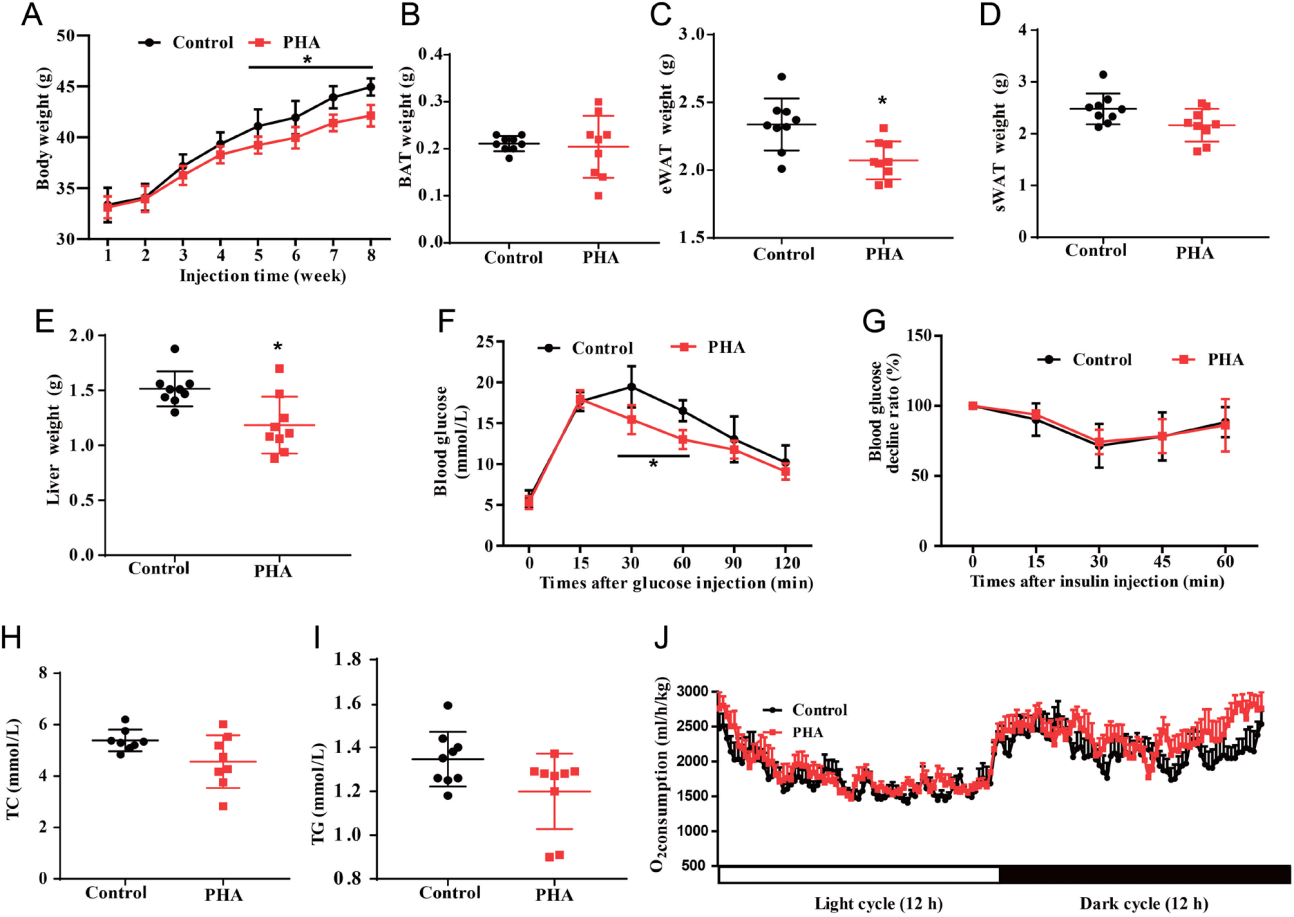
The therapeutic effects of PHA on obesity. Mice were fed continuously with a HFD for 8 weeks. Then HFD-fed obese mice were daily treated with salina or PHA (0.25 mg/kg) intraperitoneally. (A) Body weight of saline and PHA-treated HFD mice (*n* = 10 for each group) during the treatment. BAT (B), eWAT (C), sWAT (D), Liver (E) weight of saline and PHA-treated HFD mice. (F) GTT on control and PHA-treated mice (*n* = 8 for each group). (G) ITT on control and treated mice. (H) TC levels in the plasma of control and PHA treatment mice. (I) TG levels in the plasma of control and PHA treatment mice. (J) The oxygen consumption during 24 h period in HFD mice after 8 weeks of PHA treatment. A full colour version of this figure is available at https://doi.org/10.1530/JME-20-0349.

PHA prevented obesity by stimulating brown fat activity and white adipocyte browning. The therapeutic results showed that the weight gain of PHA-treated mice also decreased from the fifth week (Fig. 7A). The eWAT and liver weight of PHA-treated mice were significantly lower than that of the saline-treated group (Fig. 7C and E). There was no significant difference in BAT and sWAT (Fig. 7B, C and D). Mice treated with PHA had a better tolerance at 30 and 60 min after glucose injection (Fig. 7F). However, insulin sensitivity, oxygen consumption and serum profile had no difference in two groups (Fig. 7G, H, I and J). The therapeutic results suggest PHA also has a therapeutic effect on obesity in HFD mice.

## Discussion

Metabolic diseases such as obesity and diabetes has become a major public health concern. Recently, BAT-mediated thermogenesis was proposed as a mechanism to treat obesity and insulin resistance (Hibi *et al*. 2016). BAT transplantation reverses metabolic disorders in various obese animal models (Liu *et al*. 2015*b*). Enhanced energy expenditure by increasing BAT activity may be a promising strategy to treat obesity, diabetes, and complications due to aging. Currently, there is an intense search for bioactive compounds with anti-obesity properties, which present the particular ability to generate thermogenesis in the BAT or beige (Concha *et al*. 2019, Hui *et al*. 2020). In the study, we have referenced gene expression signatures by utilizing the microarray data obtained from eWAT in obese mice. PHA had the highest score in the CMAP database. *Phaseolus vulgaris* extract derived from the white kidney bean, previously be reported to reduce body weight, BMI, fat mass, and adipose tissue thickness (Celleno *et al*. 2007, Song *et al*. 2016), and also improved hepatic steatosis and insulin resistance by modulation of gut microbiota in HFD-fed mice (Song *et al*. 2016). A previous study showed PHA at high oral doses induced losses of body lipids because PHA reduced intestinal lipid absorption (Banwell *et al*. 1983, Pusztai *et al*. 1993). In this study, PHA was administered by intraperitoneal injection without passing through the intestinal tract. Therefore the anti-obesity effect of PHA was not mediated by impaired intestinal lipid absorption and gut microbiota. We identified PHA enhanced metabolism, limited weight gain, and ameliorated insulin resistance by increasing BAT function and inducing browning of WAT. These data suggest that the decreased body weight of PHA treatment group mice is due to high levels of energy expenditure dependent on BAT thermogenesis. However, PHA could not prevent obesity in UCP-1 KO mice induced by HFD. To our knowledge, this is the first study indicating that PHA regulates BAT function and metabolism.

A previous study showed that PHA was regarded as a nutritional toxin, but low doses of PHA reduced hyperglycemia and body fat in young growing rats (Bardocz *et al*. 1996). PHA has many physiological effects at low daily doses. Low concentrations of PHA is benefit to embryo development, but high concentrations of PHA blocks the development of embryos (Zhang *et al*. 2011). We explored the lowest working concentration of PHA, and after treatment, no morphological and functional abnormalities were found in HFD mice. We found that low doses of PHA decreased HFD-induced body weight gain due to a marked reduction in body fat mass and had no effect on the food intake, water intake and physical activity.

Compared with the control mice, PHA-treated mice showed a sizeable increase in oxygen consumption during the dark cycle (active phase), and there was no significant difference between PHA and control groups during the light cycle (non-active phase). BAT activity is positively correlated with energy expenditure and can improve glucose metabolism (Kim *et al*. 2019). BAT activity also shows circadian rhythms, with a high activity during the dark and a low activity during the light, which is regulated by a rhythmic gene family (Adlanmerini *et al*. 2019).Our results suggested that PHA could activate BAT activity without changing its circadian rhythm.

It is interesting that mice fed a HFD treated with PHA were more tolerant during the cold tolerance test but had reduced brown adipose tissue size, suggesting BAT from PHA-treated HFD mice BAT did not show accumulation of white fat and not whitening, but showed high activity. BAT contains large amounts of mitochondria and disperses lipids by UCP-1 that uncouples chemical energy to produce heat and maintain body temperature (Rachid *et al*. 2015); beige adipocytes, which resemble white adipocytes, express low UCP-1 at basal status, and have a highly inducible thermogenic capacity upon stimulation (Wu *et al*. 2012). PRDM16 plays a critical role during BAT development and is required for beige adipocyte biogenesis in WAT (Kissig *et al*. 2017). In addition, PGC-1a binds to the PPARα and PPARγ complexes plus the retinoid x receptor (RXR), activating the *Ucp1* expression through the binding to PPAR response elements in its promoter (Kajimura *et al*. 2010).We found that PHA not only increased the expression of *Ucp1* in BAT but also regulated the expression of transcription factors that participate in mitochondrial biogenesis. PHA also significantly induced *Ucp1*, *Prdm16*, *Pgc1α* expression in both BAT and WAT. These results revealed that WAT seemed to transform to beige adipose tissue within 8 weeks when HFD mice were given PHA daily. However, it will be of interest to investigate the PHA target proteins in adipose tissue, the mechanism of PHA in anti-obesity by stimulating BAT is still largely unanswered and is an active area of investigation.

## Conclusion

Altogether, the above results indicated that PHA reduced the body weight gain, maintained glucose homeostasis and improved cold tolerance through enhancing BAT activity and increased the browning of WAT. Given the ability of BAT to produce heat from stored chemical energy and thus counteract obesity, we are optimistic that PHA can be used to activate the BAT for therapeutic purposes.

## Supplementary Material

Supplementary Table 1 The sequences of primers used in real time PCR assays.

## Declaration of interest

The authors declare that there is no conflict of interest that could be perceived as prejudicing the impartiality of the research reported.

## Funding

This work was supported by the National Natural Science Foundation of P.R. China (Grants No. 81803425), the Open and Cooperation in Science and Technology Project of Henan Province (No. 182106000047), the Scientific and Technological Innovation Talent Project of Henan Province (19HASTIT015) and the Doctor Research Start-up Fund (ZKNUC2016013 and ZKNUC2016017).

## Author contribution statement

Xiaomeng Liu and ChangJing Wu designed and conceived the experiments. Yunixa Zhang, Huihui wang, Jiao Li and Yue Yu performed the experiments. Bo Li and Jin Li carried out the experiments and analyzed CMAP data. Li Huang analyzed data. Yunxia Zhang and ChangJing Wu drafted the manuscript. All authors have read the manuscript and have final approval of the submitted and published versions.
