# Supplementary material for: Phytohemagglutinin ameliorates HFD-induced obesity by increasing energy expenditure
Source: J Mol Endocrinol. 2021 May 13;67(1):1–14. doi: 10.1530/JME-20-0349 (PMC8240727; doi:10.1530/JME-20-0349)
Supplement: Supplementary Table 1 The sequences of primers used in real time PCR assays. [file supplementary_table_1.pdf]

Supplementary Table 1 The sequences of primers used in real time PCR assays.

Genes	Primer sequence(5′-3′)
Ucp1 forward	AGGCTTCCAGTACCATTAGGT
Ucp1 reverse	CTGAGTGAGGCAAAGCTGATTT
Pgc1α forward	GTCAACAGCAAAAGCCACAA
Pgc1α reverse	TCTGGGGTCAGAGGAAGAGA
Ppar1α forward	AGCCTCAGCCAAGTTGAAGT
Ppar1α reverse	TGGGGAGAGAGGACAGATGG
Prdm16 forward	CAGCACGGTGAAGCCATTC
Prdm16 reverse	GCGTGCATCCGCTTGTG
Tfarm forward	GTCCATAGGCACCGTATTGC
Tfarm reverse	CCCATGCTGGAAAAACACTT
Nrf1 forward	CAACAGGGAAGAAACGGAAA
Nrf1 reverse	GCACCACATTCTCCAAAGGT
Cyclophillin A forward	TCCAAAGACAGCAGAAAACTTTCG
Cyclophillin A reverse	TCTTCTTGCTGGTCTTGCCATTCC
